# Abiotic factors and endophytes co-regulate flavone and terpenoid glycoside metabolism in *Glycyrrhiza uralensis*

**DOI:** 10.1007/s00253-023-12441-3

**Published:** 2023-03-03

**Authors:** Zidi Liu, Yunyang Ma, Xuelian Lv, Nannan Li, Xiaohan Li, Jianmin Xing, Chun Li, Bing Hu

**Affiliations:** 1 Institute of Biochemical Engineering, College of Chemistry and Chemical Engineering, Beijing Institute of Technology, Beijing, 102401 People’s Republic of China; 2 Ningxia Academy of Agriculture and Forestry Sciences, Yinchuan, 750002 People’s Republic of China; 3 CAS Key Laboratory of Green Process and Engineering & State Key Laboratory of Biochemical Engineering, Institute of Process Engineering, Chinese Academy of Sciences, Beijing, 100190 People’s Republic of China; 4 Key Lab for Industrial Biocatalysis, Ministry of Education, Department of Chemical Engineering, Tsinghua University, Beijing, 100084 People’s Republic of China; 5 Key Laboratory of Medical Molecule Science and Pharmaceutical Engineering, Ministry of Industry and Information Technology of China, Beijing, 102401 People’s Republic of China

**Keywords:** *Glycyrrhiza uralensis* Fisch., Secondary metabolite synthesis, Root-associated endophytes, 16S rDNA, Transcriptomics

## Abstract

**Abstract:**

Recently, endorhizospheric microbiota is realized to be able to promote the secondary metabolism in medicinal plants, but the detailed metabolic regulation metabolisms and whether the promotion is influenced by environmental factors are unclear yet. Here, the major flavonoids and endophytic bacterial communities in various *Glycyrrhiza uralensis* Fisch. roots collected from seven distinct places in northwest China, as well as the edaphic conditions, were characterized and analyzed. It was found that the soil moisture and temperature might modulate the secondary metabolism in *G. uralensis* roots partially through some endophytes. One rationally isolated endophyte *Rhizobium rhizolycopersici* GUH21 was proved to promote the accumulation of isoliquiritin and glycyrrhizic acid significantly in roots of the potted *G. uralensis* under the relatively high-level watering and low temperature. Furthermore, we did the comparative transcriptome analysis of *G. uralensis* seedling roots in different treatments to investigate the detailed mechanisms of the environment-endophyte-plant interactions and found that the low temperature went hand in hand with the high-level watering to activate the aglycone biosynthesis in *G. uralensis*, while GUH21 and the high-level watering cooperatively promoted the *in planta* glucosyl unit production. Our study is of significance for the development of methods to rationally promote the medicinal plant quality.

**Key points:**

*• Soil temperature and moisture related to isoliquiritin contents in Glycyrrhiza uralensis Fisch.*

*• Soil temperature and moisture related to the hosts’ endophytic bacterial community structures.*

*• The causal relation among abiotic factors—endophytes—host was proved through the pot experiment.*

**Supplementary Information:**

The online version contains supplementary material available at 10.1007/s00253-023-12441-3.

## Introduction

Many plant roots are involved in the pharmaceutical and food industries due to their abundances of secondary metabolites with high pharmacological activities, such as ginseng (Riaz et al. 2019) and licorice (Jiang et al. 2020). Considering that the over-excavation of wild resources is causing habitat destruction and biodiversity wreck, people have started the domestic cultivation of medicinal plants to meet the increasing demand for their roots (Canter et al. 2005; Li et al. 2015). However, the annual yield of medicinal products originated from the roots of the cultivated species are generally limited, because medicinal plants are often difficult to breed (Wang et al. 2020a, b) and their roots’ quality is relatively low compared with the wild resources in general (Murthy et al. 2018; Wang et al. 2018). Besides with the plant genotypes (Yuan et al. 2010; Wang et al. 2020a, b), climatic characteristics (Li et al. 2020a, b), soil physiochemical properties (Miransari et al. 2021), and growth years (Bai et al. 2020), soil microbiomes (Shi et al. 2011) are expected to be significantly associated with the abundances of the bioactive ingredients in roots. Soil microbes are known to be actively recruited via root exudates (Sasse et al. 2018) and grow on plant-derived metabolites at the endorhizosphere. In return, some endorhizospheric microbes, namely endophytes, could improve their hosts’ growth, pathogen resistance, and abiotic stress tolerance during their symbiosis (Wani et al. 2015; Mukherjee et al. 2021), while some of them could stimulate the *in planta* signal transduction-induced secondary metabolism of their host plants for the production of pharmaceutically important compounds (Korenblum and Aharoni 2019; Ahmed and Hijri 2021; Chen et al. 2021). Pandey et al. (2018) found that, compared with the endophyte-free *Withania somnifera*, the ones inoculated with some endophytic individuals, being isolated from the leaves, roots, and seeds of *W. somnifera* and being unable to produce withanolides alone, had significantly higher contents of the pharmaceutically active steroidal lactones 12-deoxy withstramonolide and withanolide A in roots after a 3-month cultivation, which was probably due to the endophyte-associated increasing of *in planta* indole 3-acetic acid (IAA). Although certain advances have been made in medicinal plant-endophyte interaction, there remain a number of important questions in the direction. Accordingly, secondary metabolite production-promoting endophytes are obviously less reported than soil microbes with other functions (Mariotte et al. 2018), and thus the detailed mechanisms of endophyte-induced plant secondary metabolism are not declared yet. Meanwhile, considering that the plant–microbe symbiosis is significantly impacted by environmental conditions (Cheng et al. 2019), the performances of secondary metabolite production-promoting endophytes in certain plants are inferred to be affected by various environmental factors, but it is seldomly investigated yet.

*Glycyrrhiza uralensis* Fisch, which is a salt- and drought-tolerant legume, is natively grown or artificially planted in arid to semi-arid sandy soil in and around central Asia (Jiang et al. 2020). Its roots are rich in pharmaceutically active flavonoids, some of which are unique to licorice (Zhou et al. 2020). For example, ILQ, a member of chalcone glycosides, is practically applied on various disease preventions and treatments due to its antidepressant, anticancer, antioxidative, and anti-inflammatory activities (Zhou et al. 2017). The flavonoid contents in licorice are significantly influenced by the environmental factors (e.g., soil salt content, soil moisture, and sunshine duration) through the stress-induced phytohormone signaling pathways (Yu et al. 2015; Xie et al. 2018; Wang et al. 2021). Meanwhile, the endophyte-free *G. uralensis* roots were found to have significantly lower contents of bioactive components than the normal ones after a 3-month pot cultivation (Yu et al. 2020), suggesting that endophytic microbiota could regulate the accumulation of secondary metabolites in licorice roots. Interestingly, the endophytic microbiota compositions were also significantly influenced by the environmental factors, as well as the content of total flavonoids in licorice roots (Dang et al. 2020). Therefore, the flavonoid biosynthesis and accumulation in *G. uralensis* is inferred to be driven by the environment-endophyte-plant interactions (Liu et al. 2021), but the detailed mechanisms are undeclared yet.

Currently, there are few root-associated soil microbes with the function to promote root-associated secondary metabolite production in *G. uralensis* (Chen et al. 2017; Xie et al. 2018), and how endophytes promote the secondary metabolite accumulation in *G. uralensis* roots under different environmental variables is not clear. In the study, we collected 42 *G. uralensis* root samples from seven distinct places in northwest China and did the systematically bioinformatic analyses on the environmental factors of the sampling sites, root-associated flavonoid profiles, and the root-associated endophytic community compositions. Based on the Spearman correlation analyses, as well as the conventional microbe screening, we rationally screened out a flavonoid accumulation-promoting endophytic bacterium candidate *Rhizobium rhizolycopersici* GUH21 from the *G. uralensis* root samples. The pot experiments of *G. uralensis* seedlings with or without *R. rhizolycopersici* GUH21 under different environmental conditions confirmed the bioinformatics prediction and elucidated the molecular mechanism of the metabolic promotion. The isolation and deeply understanding of plant secondary metabolite-promoting endophytes would be significant for the development of techniques in agriculture and pharmaceutical manufacturing industry.

## Materials and methods

### Sampling of *Glycyrrhiza uralensis* Fisch. roots and ectorhizospheric soil

Triplicated *G. uralensis* roots and the responding ectorhizospheric soil samples were collected from 14 sites in seven distinct places in the northwest China, including Hangjinqi (HJQ) in Inner Mongolia Province, Yanchi (YC) and Hongsipu (HSP) in Ningxia Province, and Minqin (MQ), Jiuquan (JQ), Yuzhong (YZ), and Zhangye (ZY) in Gansu Province. Some samples that were from the wild *G. uralensis* were named as “location abbreviation-W,” the samples that were from the 3-year-old-cultivated *G. uralensis* were named as “location abbreviation-C,” the samples that were from the 7-year-old-cultivated *G. uralensis* were named as “location abbreviation-C7,” and the samples that were from the 9-year-old-cultivated *G. uralensis* were named as “location abbreviation-C9.” The names of all 14 groups and their geographical positions are as follows: HJQ-C (40.78̊N, 108.29 °E), HJQ-W (40.78̊N, 108.32 °E), YC-C (37.81 °N, 107.28 °E), YC-W (36.16 °N, 104.32 °E), HSP-C (37.38 °N, 106.08 °E), HSP-W (37.38 °N, 106.10 °E), JQ-C (40.15 °N, 99.06 °E), JQ-W (40.15 °N, 99.04 °E), MQ-C (38.55 °N, 102.66 °E), MQ-C7 (38.54 °N, 102.69 °E), YZ-C (36.16 °N, 104.32 °E), YZ-W (36.16 °N, 104.32 °E), ZY-C9 (38.85 °N, 100.65 °E), and ZY-W (38.85 °N, 100.66 °E). The roots were washed by clean water, 75% (v/v) ethanol, and sterilized water successively soon after they were digged up. Each group of root samples was divided into two parts for the storage: one part was kept at 4 °C for endophyte isolation, and the other part was frozen with dry ice for endophytic microbial community sequencing and bioactive ingredient determination. The ectorhizospheric soil samples were put in the sampling bags and then kept on ice for the further operation.

### Determination of the edaphic parameters at the sampling sites

The in situ soil temperature was measured at the sampling sites using a soil thermometer TP101 (Haofei Instrument Technology Co., Ltd., Dongtai, P.R. China) soon after the digging processes. The other seven edaphic parameters of the sampling sites, including the soil moisture, pH, saltiness, total carbon content (TC), total nitrogen content (TN), organic carbon content (OC), and TC/TN, were measured in the lab as described in Methods S1.

### Analysis of root-associated secondary metabolites

The frozen *G. uralensis* root samples were dried in a vacuum freeze drier (Biocool, Beijing, P.R. China) and ground into powder using the mortar. The soluble ingredients in the powders were extracted by 100 mL of 70% (v/v) ethanol. The target compounds in the extracted solutions, including liquiritigenin (LG), isoliquiritigenin (ILG), isoliquiritin (ILQ), licochalcone A (LCA), neohesperidin dihydrochalcone (NHDC), and glycyrrhizic acid (GL), were qualified via the high-performance liquid chromatography (HPLC 1260 Infinity; Agilent, Santa Clara, CA, USA) according to Methods S2. To quantify these metabolites, a mixture of 0.28 g/L LG, 0.28 g/L ILG, 0.28 g/L ILQ, 0.28 g/L LCA, 0.28 g/L NHDC, and 4.08 g/L GL purchased from Shanghai yuanye Bio-Technology Co., Ltd (Shanghai, China) was utilized as the HPLC original standard solution.

### Root-associated endophytic bacterial community sequencing

The DNA in frozen *G. uralensis* root tissues (~ 100 mg each) was extracted using a FastDNA® Spin Kit for Soil (MP Biomedicals, Santa Ana, CA, USA) following the manufacturer’s introduction. The DNA concentration and quality were estimated using a NanoDrop spectrophotometer (Thermo Fisher Scientific, Waltham, MA, USA) and the electrophoresis in 1% (w/v) agarose gel. The conserved V3-V4 region of the 16S rRNA gene from the qualified DNA samples was amplified by using the primer pair 338F (5′-ACTCCTACGGGAGGCAGCAG-3′) and 806R (5′-GGACTACHVGGGTWTCTAAT-3′). The thermal cycling process of the polymerase chain reaction (PCR) is as follows: 95 °C for 3 min; 27 cycles each of which contained the denaturation at 95 °C for 30 s, annealing at 55 °C for 30 s, and extension at 72 °C for 45 s, 72 °C for 10 min; 4 °C forever. The PCR products were extracted from 2% agarose gel and purified using the AxyPrep DNA Gel Extraction Kit (Axygen Biosciences, Union City, CA, USA) according to manufacturer’s instructions and then quantified using the Quantus™ Fluorometer (Promega, Madison, WI, USA).

The purified PCR products were loaded on an Illumina MiSeq PE300 platform (Illumina, San Diego, USA) for DNA fragment sequencing according to the standard protocols by Majorbio Bio-Pharm Technology Co. Ltd. (Shanghai, China). The raw 16S rDNA sequencing reads were demultiplexed, quality-filtered by fastp v 0.20.0 (Chen et al. 2018), and merged by FLASH v1.2.7 (Magoc and Salzberg 2011) with the following criteria: (1) the 300 bp reads were truncated at any site receiving an average quality score of < 20, and the truncated reads shorter than 50 bp and reads containing ambiguous characters were discarded; (2) only overlapping sequences longer than 10 bp with the overlap region mismatching ratio ≤ 0.2 were assembled according to their overlapped sequence, and reads that could not be assembled were discarded; (3) sequences and their directions of each samples were distinguished according to the barcode and primers, and reads containing more than two nucleotide mismatch in primer matching were discarded. Clustering of operational taxonomic units (OTUs) with 97% similarity cutoff and the chimeric sequence filtering were simultaneously performed on UPARSE version 7.1 (http://drive5/com/uparse). The taxonomy of each OTU representative sequence was analyzed via RDP Classifier version 2.2 (Wang et al. 2007) against the 16S rDNA sequence Silva database (Quast et al. 2012) using the confidence threshold of 0.7.

### Isolation and identification of endophytes from *G. uralensis* Fisch. roots

The *G. uralensis* root samples were kept at 4 °C and soaked in 3% (v/v) sodium hypochlorite solution for 5 min to radically kill microbes on root surface. The surface-clean roots were washed with the sterilized double distilled water (ddH_2_O) for three times and then mashed in a sterilized blender. The mashed root tissues were suspended in 10 mL 0.9% (w/v) NaCl solution and filtered through sterile gauze. The filtrates were plated on different solid media as listed in Table S1 and then cultivated at room temperature to originally isolate the proposed root-associated endophytes. The developed colonies showing different morphological appearance were selected and purified on a new plate containing the same media. The classifications of the purified isolates were tentatively identified based on their 16S rDNA sequences according to the standard procedure of Yanagi and Yamasato (1993). The identified endophytic strain *R. rhizolycopersici* GUH21 was deposited into the Chinese General Microbiological Culture Collection Center (CGMCC) with the accession number of 24,152.

### The pot experiments

One hundred and twenty *G. uralensis* seeds were successively soaked in 70% (v/v) ethanol for 10 min and 10% (v/v) hymexazol (C_4_H_5_NO_2_) for 2 h to kill microbes on the seeds according to the European Food Safety Authority (European 2015) and Egamberdieva et al. (2021). The sterile seeds were evenly planted in 24 pots each containing 0.5 kg sterilized organic active matrix (Ningxia Zhongqing Biotechnology, Ningxia, P.R. China) and 300 mL sterilized ddH_2_O, and then the pots were kept at 26 °C and 50% humidity until the germination (about one week). Soon after the tender buds could be observed, the seedlings in each of 12 pots were inoculated with 65 mL culture suspension of the plant secondary metabolite production-promoting endophytic candidate (OD_600_ = 1.0), while the seedlings in the other 12 pots were irrigated with 65 mL sterilized ddH_2_O and termed as the controls (CKs). The 24 pots were kept at either low- or high-temperature (21 or 26 °C) and watering amounts (ddH_2_O 270 or 330 mL per pot once a week (mL/pot/we)) for 60 days. The experimental conditions for each treatment are listed in Table 1.

**Table 1:** The designed pot experiment for *Glycyrrhiza uralensis* Fisch. to identify the effects of endophyte candidates and key abiotic factors

Treatment label	Group^a^	Endophyte inoculum	Temperature (°C)	Watering amount (mL/pot/we)
21L	CK	-	21	270
	GUH21	*R. rhizolycopersici* GUH21		
21H	CK	-	21	330
	GUH21	*R. rhizolycopersici* GUH21		
26L	CK	-	26	270
	GUH21	*R. rhizolycopersici* GUH21		
26H	CK	-	26	330
	GUH21	*R. rhizolycopersici* GUH21		

^a^Each group had triplicates

After the 60-day cultivation, the *G. uralensis* plants in each pot were dug up and were divided into two copies. One copy was used to determine the morphological characteristics of the harvested plants, including the dry weights of the aboveground and underground parts, the area of the apical leaves and the lateral leaves, the root lengths, and the root diameters, while the other copy was frozen with dry ice for RNA extraction and transcriptomics analysis as described in the next section. The fresh roots after the samples’ morphological characteristics determination were frozen dried and processed to determine the contents of ILQ and GL according to the methods described in the previous section.

### Transcriptome sequencing of the cultivated *G. uralensis* Fisch. roots

RNA from a weight of 50 mg surface-cleaned and frozen *G. uralensis* roots in each treatment was extracted using TRIzol™ Plus RNA Purification Kit for plant tissues (Thermo Fisher Scientific, Waltham, MA, USA) according to the manufacturer’s instructions and was purified using DNase I (TaKara Biomedical Technology Co., Ltd., Beijing, P.R. China). The RNA quality was determined by 2100 Bioanalyser (Agilent, Santa Clara, CA, USA) and quantified by the NanoDrop spectrophotometer ND-2000. Afterwards, 1 μg of high-quality total RNA was processed using TruSeqTM RNA sample preparation Kit (Illumina, San Diego, CA, USA), and then messenger RNA (mRNA) was isolated according to poly A selection method by oligo(dT) beads, fragmented by fragmentation buffer, and then reverse transcribed using a SuperScript double-stranded cDNA synthesis kit (Invitrogen, Carlsbad, CA, USA) with random hexamer primers (Illumina) to form the double-stranded cDNAs. The synthesized cDNA was subjected to end-repair, phosphorylation, and “A” base addition according to Illumina’s library construction protocol. Libraries were size selected for cDNA target fragments of 300 bp. The paired-end cDNA library was sequenced with the Illumina HiSeq xten/NovaSeq 6000 sequencer. The raw reads were trimmed by SeqPrep (https://github.com/jstjohn/SeqPrep) and Sickle (https://github.com/najoshi/sickle) with default parameters. Then clean reads were separately aligned to reference genome with orientation mode using HISAT2 (Kim et al. 2019). The mapped reads of each sample were assembled by StringTie (Pertea et al. 2015). In addition, functional-enrichment analysis including Clusters of Orthologous Genes (COG) and Kyoto Encyclopedia of Genes and Genomes (KEGG) annotation were carried out by BLASTp to the COG database (https://www.ncbi.nlm.nih.gov/research/COG) and KOBAS 3.0 (Xie et al. 2011), respectively.

### Statistical analysis

Shapiro–Wilk normality test was utilized to determine if data had a normal distribution. The Student’s *t*-test performed using the *t.test* function in Microsoft Excel Office2019 (Microsoft, Redmond, WA, USA) was applied to determine the significance of the differences between two groups. The microbial diversity analyses of the root-associated endophytic bacterial communities and the transcriptome difference analysis of the cultivated *G. uralensis* roots were performed using the R Project for Statistical Computing version 4.1.2. The alpha diversity (Shannon–Wiener indices and Simpson indices) of the genus-level microbiota were estimated using the *shannon* < *-diversity* and *simpson* < *-diversity* functions, respectively, in the *vegan* package of R, while the beta diversity in terms of the Bray–Curtis dissimilarity was calculated using the *vegdist* function. The principal component analysis (PCA) and redundancy analysis (RDA) were performed using the *rda* function in the *vegan* package of R, respectively. The Spearman’s rank correlation was calculated using the *cor* function in the *stats* package of R. For the transcriptomics analysis, the identification of the differentially expressed genes (DEGs) between two treatments was performed using the DESeq2 package v 1.34.0 (Love et al. 2014) in R, wherein the genes with the adjusted *p*-value < 0.05 and the |log_2_(fold change)|≥ 1 were considered to be DEGs.

## Results

### Differentiation of root-associated flavonoid profiles relating to *in situ* abiotic environmental factors

In the study, 42 *G. uralensis* roots, as well as the rhizospheric soil, were sampled in June, 2020, from seven distinct places in northwest China, including HJQ, YC, HSP, MQ, JQ, YZ, and ZY. Excepting for the ones collected from MQ where no wild *G. uralensis* but the 7-year-cultivated *G. uralensis* (named as MQ-C7) were found, and the ones from ZY where no 3-year-cultivated *G. uralensis* but the 9-year-cultivated *G. uralensis* (named as ZY-C9) were available, the root samples in each site contained both wild and 3-year-cultivated *G. uralensis* and were named as “location abbreviation-W” and “location abbreviation-C”, respectively.

**Fig. 1:**
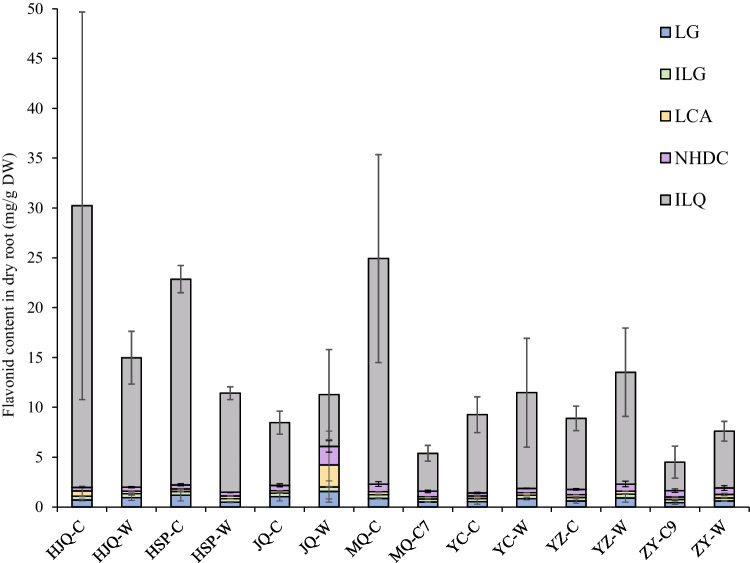
The contents of five flavonoids in *Glycyrrhiza uralensis* roots collected from distinct places in northwest China. ###-W, wild *G. uralensis* collected from ###; ###-C, 3-year-cultivated *G. uralensis* collected from ###; ###-C7, 7-year-cultivated *G. uralensis* collected from ###; ###-C9, 9-year-cultivated *G. uralensis* collected from ###; LG, liquiritigenin; ILG, isoliquiritigenin; ILQ, isoliquiritin; LCA, licochalcone A; NHDC, neohesperidin dihydrochalcone

Contents of five major flavonoids in each *G. uralensis* root sample, including liquiritigenin (LG), isoliquiritigenin (ILG), isoliquiritin (ILQ), licochalcone A (LCA), and neohesperidin dihydrochalcone (NHDC), were determined. As shown in Fig. 1, the inference that wild *G. uralensis* should have higher contents of root-associated bioactive compounds than the cultivated species was not suitable in our case, and the planting conditions were considered to be much more suitable than the wild environment for the flavonoid accumulation in *G. uralensis* in some places, such as HJQ and HSP. On the other hand, ILQ was the predominant root-associated flavonoid in all samples, ranging from 2.848 ± 1.606 mg/g in ZY-C9 to 28.267 ± 19.459 mg/g in HJQ-C, but its content was significantly more varied than the other four flavonoids LG, ILG, LCA, and NHDC. This indicated that ILQ synthesis metabolism was always more active and more easily influenced by environmental factors than the metabolic pathways of LG, ILG, LCA, and NHDC in *G. uralensis*,

To investigate the significances of abiotic environmental factors for *in planta* ILQ accumulation, the in situ edaphic conditions of the sampling sites, including soil temperature, pH, saltiness, moisture, TC, OC, TN, and TC/TN were measured (Table S2) and co-analyzed with the flavonoid profiles using the linear regression (Table 2). Compared with other edaphic variables, the soil moisture had the highest absolute correlation coefficient with ILQ (*r* = 0.3541), followed by the soil temperature (*r* =  − 0.2302). The linear equations reflected soil moisture had the positive association with ILQ content, and soil temperature had the negative correlation with ILQ content. Therefore, it was inferred that soil moisture and temperature should be the most important factors for root-associated ILQ accumulation, and the higher the moisture content and the lower the temperature within limits, the higher ILQ content was in *G. uralensis* roots.

**Table 2:** Linear regression analysis of the edaphic conditions and root-associated liquiritigenin (ILQ) content in sampling sites

Independent variable	Linear equation^e^	Correlation coefficient r
Temperature (°C)	*y* = − 0.0005x + 0.0192	− 0.2302
pH	*y* = 0.0031x − 0.0153	0.1616
Saltiness (mg/kg)^a^	*y* = 0.0043x + 0.0099	0.0045
Moisture (%)	*y* = 0.0006x + 0.0043	0.3541
TC (mg/g)^b^	*y* = 0.2377x + 0.0058	0.1982
OC (mg/g)^c^	*y* = 0.273x + 0.0061	0.2074
TN (mg/g)^d^	*y* = 6.1667x + 0.0079	0.1844
TC/TN	*y* = − 2E-05x + 0.0111	− 0.1797

^a^Saltiness, salt content in dry soil

^b^TC total carbon content in dry soil

^c^*OC* organic carbon content in dry soil

^d^TN total nitrogen content in dry soil

^e^Isoliquiritin content in dry root (mg/g) as the dependent variable

### Differentiation of root-associated endophytic bacterial communities relating to abiotic variables

**Fig. 2:**
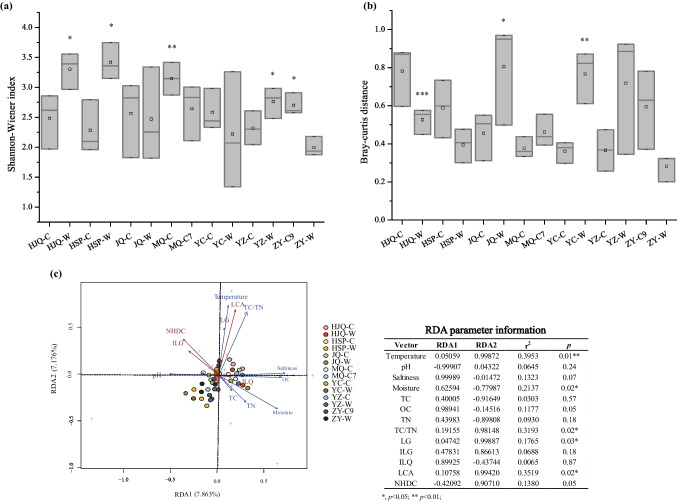
Analysis of the root-associated endophytic bacterial community structures in the wild and cultivated *G. uralensis* from different places. **a** The box plot showing the Shannon–Wiener indices of the genus-level endophytic bacterial communities in each group; **b** the box plot showing the Bray–Curtis distances among the genus-level endophytic bacterial community triplicates in each group; **c** the redundancy analysis (RDA) plot showing the influences of edaphic conditions (red) and root-associated flavonoid contents (purple) on the root-associated endophytic bacterial community compositions. The asterisk on top of boxes represents the differential significance of indices between the responding sample and ZY-W in Student’s *t*-test. *, *p* < 0.05; **, *p* < 0.01; ***, *p* < 0.005

According to the 16S rDNA amplicon sequencing, the endophytic bacterial communities in the 42 root samples contained 9,689,062 ~ 23,449,241 counts of amplicon sequences. The sequences were arranged into 696 genera and 2203 operational taxonomic units (OTUs) with a farthest neighbor jukes-Cantor distance of 0.03. In the genus level, *Amycolatopsis*, *Steroidobacter*, *Caulobacter*, and unclassified genera occupied ~ 70% of the endophytic bacterial communities in general, but the detailed compositions varied dramatically among samples (Fig S1a). As shown in Fig. 2a, samples in ZY-W had relatively lower Shannon–Wiener index (*H* = 2.18 ± 1.99) than the other ones, especially lower than that in HJQ-W, HSP-W, MQ-C, YZ-W, and ZY-C9 (*p* < 0.05). Similar results were obtained by using Simpson’s diversity index (Fig S1b). For the beta diversity analysis, the Bray–Curtis distances among the triplicates in ZY-W (BC = 0.28 ± 0.07) were relatively lower than the other ones, but the differences between ZY-W and other samples were not significant (*p* > 0.05) excepting for HJQ-W, JQ-W, and YC-W (Fig. 2b). These results indicated that the triplicates in ZY-W were differentially simple in the endophytic bacterial community composition, while the root-associated endophytic bacteria in HSP-W, MQ-C, YZ-W, and ZY-C9 samples were similarly abundant. Interestingly, this was partially in accordance with the flavonoid composition results in Fig. 1, wherein roots in ZY-W had relatively lower contents of ILQ than those in HSP-W, MQ-C, and YZ-W, suggesting that endophytic bacterial community diversities might have the tight association with ILQ accumulation in *G. uralensis* in some places.

The PCA based on the genus-level endophytic bacterial community compositions showed that samples from adjacent locations tended to be clustered together (Fig S1c), indicating that environmental conditions played an important role in the assemblage of the root-associated endospheric bacterial communities. Therefore, the influences of the in situ edaphic variables and in vivo root-associated flavonoid contents on endophytic bacterial community compositions were evaluated by using RDA. In the RDA plot (Fig. 2c), with 7.863% accountable variance for the first major principal component RDA1 and 7.176% for the second major principal component RDA2, the most significant edaphic factor was soil temperature (r^2^ = 0.40, *p* = 0.01), followed by TC/TN (r^2^ = 0.32, *p* = 0.02) and soil moisture (r^2^ = 0.21, *p* = 0.02), and the most significant flavonoids was LCA (r^2^ = 0.34, *p* = 0.02). These results suggested that the root-associated endophytic bacterial communities in *G. uralensis* were significantly influenced by both edaphic conditions and the endospheric flavonoids.

### Isolation and identification of the ILQ-relating endophytic bacterium based on the environment-endophyte-plant interactions

**Fig. 3:**
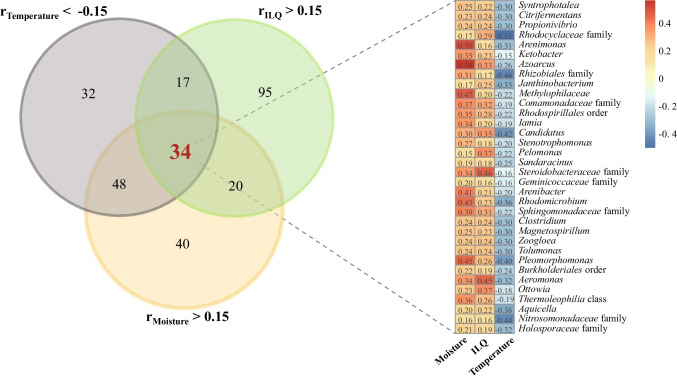
The integrated Venn diagram and correlation plot showing *G. uralensis* root-associated ILQ production-promoting endophytic species candidates which had the negative Spearman’s correlation coefficients (blue) with soil temperature and the positive correlation coefficients (red) with soil moisture and ILQ

Since the edaphic factors of soil moisture and soil temperature influenced both the root-associated ILQ accumulation and endophytic bacterial community compositions according to above findings, it was inferred that the influences of the soil moisture and temperature on the root-associated ILQ accumulation were partially performed through the endophytes. To identify the endophytic members which might be the connection between environmental factors and *G. uralensis* ILQ metabolism, a Spearman rank correlation analysis between the 42 endophytic bacterial communities and each of the three abiotic factors, including the soil moisture, the soil temperature, and the root-associated ILQ contents, were processed. As shown in Fig. 3, there were 166 endophytic genera showing the positive correlation with the ILQ contents (r_ILQ_ > 0.15), among which 51 genera had the negative relationship with the soil temperature (r_temperature_ <  − 0.15), 54 genera had the positive relationship with the soil moisture (r_moisture_ > 0.15), and 34 genera had both the negative relationship with the soil temperature and the positive relationship with the soil moisture. These 34 genera were expected to be the ones responding for *in planta* ILQ accumulation through the positive control of the soil moisture and the negative control of the soil temperature.

In order to obtain the endophytic strains of the 34 genera, fresh *G. uralensis* root samples were processed and placed on different culture media containing a slight amount of licorice root powder (Table S1). Dozens of microbial strains were isolated, and one of them was involved in the listed bacteria in Fig. 3. The strain was identified as *Rhizobium rhizolycopersici* in *Rhizobiales* family based on its 16S rRNA sequence, with the name GUH21, and it was isolated from the HJQ-C root sample. The 16S rRNA gene sequence of *R. rhizolycopersici* GUH21 was submitted to GenBank (www.ncbi.nlm.nih.gov) with accession number OQ338369.1.

### The pot experiment showing the integrated effects of abiotic and biotic factors on *G. uralensis* growth and root-associated secondary metabolite contents

**Fig. 4:**
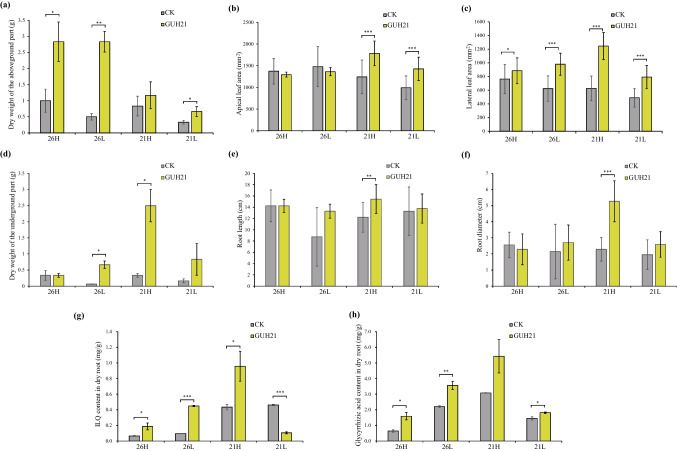
Histograms showing the physiological and biochemical characteristics of the *G. uralensis* plant after a 60-day pot experiment. 26H, plant grown at 26 °C with 330 mL/pot/we watering amount; 26L, plant grown at 26 °C with 270 mL/pot/we watering amount; 21H, plant grown at 21 °C with 330 mL/pot/we watering amount; 21L, plant grown at 21 °C with 270 mL/we watering amount; CK, plant with the addition of ddH_2_O at the beginning of the experiment; GUH21, plant inoculated with *Rhizobium rhizolycopersici* GUH21 at the beginning of the experiment. The sample size of each group in **a**, **d**, **g**, and **h** was *n* = 3, respectively; The sample size of each group in **b**, **e**, and **f** was *n* = 10, respectively; the sample size of each group in **c** was *n* = 20. The data set in each group had a normal distribution according to the Shapiro–Wilk normality test. *, *p* < 0.05 in Student’s *t*-test, **, *p* < 0.01 in Student’s *t*-test; ***, *p* < 0.005 in Student’s *t*-test

To confirm the combined effects of *R. rhizolycopersici* GUH21 and key abiotic factors (i.e., soil moisture and temperature) on ILQ accumulation in *G. uralensis*, *G. uralensis* seedling pot experiments were performed as described in Table 1. As shown in Fig. 4a–c, the leaves and aerial stems of *G. uralensis* in *R. rhizolycopersici* GUH21 inoculated groups generally grew much better than those without the inoculation (CK), regardless of the cultivation conditions (*p* > 0.05). For the underground parts, compared with the CK groups, *R. rhizolycopersici* GUH21 significantly improved the rhizome biomass accumulation (Fig. 4d), root elongation (Fig. 4e), and root thickening (Fig. 4f) in the 21H groups, wherein the temperature was 21 °C and the watering amount was 330 mL/pot/we. Meanwhile, the bioactive ingredients in the roots were measured after the harvesting. As shown in Fig. 4g, the ILQ contents in CK roots in 21H (0.434 ± 0.034 mg/g) and 21L (0.462 ± 0.009 mg/g), wherein the temperature was 21 °C and the watering amount was 330 and 270 mL/pot/we, respectively, were significantly higher than those in 26H (0.066 ± 0.006 mg/g) and 26L (0.095 ± 0.003 mg/g), wherein the temperature was 26 °C. This suggested that *G. uralensis* roots preferred to accumulate ILQ under the relatively low temperature. The promotion of low temperature was strengthened by *R. rhizolycopersici* GUH21 at the relatively high-watering amounts in 21H (0.958 ± 0.191 mg/g) but severely weakened by the same species at the relatively low-watering amounts in 21L (0.105 ± 0.013 mg/g). For *G. uralensis* grown at 26 °C, the contents of root-associated ILQ in treatments with *R. rhizolycopersici* GUH21, ranging from 0.142 to 0.455 mg/g, were significantly higher than those in CK, no matter the watering amount was high (26H) or low (26L). These results indicated that *R. rhizolycopersici* GUH21 had the capability to elicit the *in planta* ILQ accumulation in *G. uralensis* roots, and the performance was enhanced under the relatively low temperature and relatively high-level watering. Interestingly, glycyrrhizic acid (GL), the major terpenoid in *G. uralensis* roots, also increased in *R. rhizolycopersici* GUH21 inoculated groups compared with that in the CK groups, especially under the cultivation condition in the 21H group (Fig. 4h). These results suggested that *R. rhizolycopersici* GUH21 could help *G. uralensis* accumulate not only flavonoids but also terpenoids, and the abiotic and biotic factors performed the synergistic effects on root-associated secondary metabolite accumulation, which was in accordance with the previous findings in Table 2 and Fig. 3.

### Transcriptome analyses on the 60-day cultivated *G. uralensis* roots

**Fig. 5:**
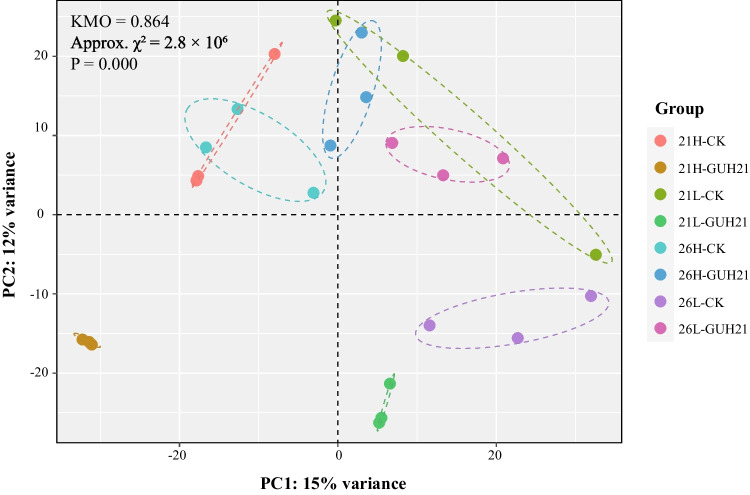
The principal component analysis (PCA) plot of the normalized transcriptome count data sets in the 60-day-old *G. uralensis* roots cultivated under different abiotic and biotic conditions. ##-CK, treatment without *R. rhizolycopersici* GUH21 inoculum; ##-GUH21, treatment with the addition of *R. rhizolycopersici* GUH21. KMO, Kaiser–Meyer–Olkin measure of sampling adequacy; Approx. χ^2^ and P, approximate Chi-square and the significance of count data, respectively, through the Bartlett’s test of sphericity

To investigate the metabolic regulation of *R. rhizolycopersici* GUH21 and abiotic environmental factors on *in planta* ILQ and GL production, the comparative transcriptome analyses of high-throughput mRNA sequencing (RNA-seq) data sets profiling gene expression in the *G. uralensis* roots of each group were operated. The PCA plot, wherein the RNA-seq count data were normalized using the *rlog* function in the DESeq2 package, reflected that the transcriptomes of *R. rhizolycopersici* GUH21-inoculated roots in the 21H group (21H-GUH21) were clearly separated from those of other roots and so was the RNA-seq data of *R. rhizolycopersici* GUH21-inoculated roots in 21L (21L-GUH21), suggesting that *R. rhizolycopersici* GUH21 inoculation and the cultivation temperature greatly influenced the gene expression in *G. uralensis* roots (Fig. 5). Meanwhile, because the RNA-seq data of CK roots in 21H (21H-CK) and 26H (26H-CK) were clustered in the beta quadrant of the PCA plot and clearly separated from the CK roots in 21L (21L-CK) and 26L (26L-CK), it was considered that the gene expression in *G. uralensis* roots was also influenced by the water supply.

**Fig. 6:**
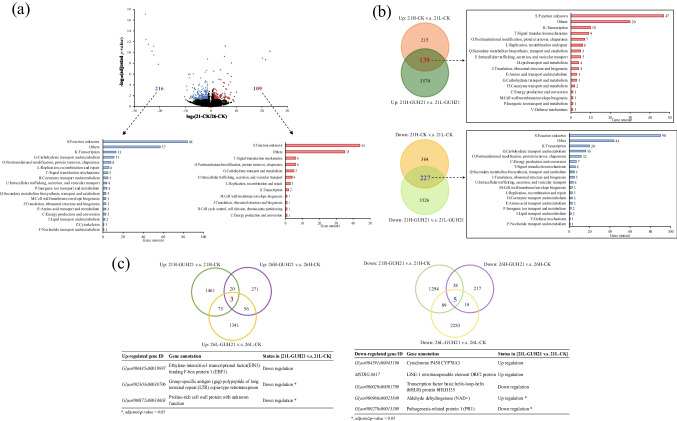
Identification of differential gene expression in the 60-day-old *G. uralensis* roots cultivated under different abiotic and biotic conditions. **a** The volcano plot showing the differentially expressed genes (DEGs) between the CK roots cultivated under 21 °C (CK-21) and 26 °C (CK-26), wherein the red dots represented genes whose expression abundances were significantly higher in CK21 than those in CK26 (fold change ≥ 2 and adjusted *p*-value < 0.05 in DESeq2) and whose COG functional categories were listed in the bottom right red bar chart, and the blue dots represented genes whose expression abundances were significantly lower in CK21 than those in CK26 (fold change ≤ 0.5 and adjusted *p*-value < 0.05 in DESeq2) and whose COG functional categories were listed in the bottom left blue bar chart. **b** The Venn diagrams showing the expressed genes being significantly higher (above) or lower (bottom) in 21H-CK and 21H-GUH21 than those in 21L-CK and 21L-GUH21, respectively, wherein the bold red number in the above diagram represented the amount of DEGs being significantly higher in both 21-CK/21L-CK and 21H-GUH21/21L-GUH21 and their COG functional categories were on the right red bar chart, and the bold blue number in the bottom diagram represented the DEGs being significantly lower in both comparison groups, and their COG functional categories were on the right blue bar chart. **c** The Venn diagrams showing expressed genes being significantly higher (left) or lower (right) in 21H-GUH21, 26H-GUH21, and 26L-GUH21 than those in their responding CK groups, wherein the bold red number in the left diagram represented the amounts of up-regulated genes being shared by the three comparison groups and their gene annotation, as well as their relative contents in 21L-GUH21/21L-CK, were listed in the bottom left table, and the bold blue number in the right diagram represented the amounts of down-regulated genes being shared by the three comparison groups, and their gene annotations and relative contents in the 21L comparison group were listed in the bottom right table

To identify the detailed influences of temperature, watering, and *R. rhizolycopersici* GUH21 on gene expression in *G. uralensis* roots, differential expression gene analyses were performed using DESeq2. As shown in Fig. 6a, to explain why root-associated ILQ accumulated more at the relatively low temperature, the transcriptome comparison between CK roots cultivated under 21 °C (CK-21) and those under 26 °C (CK-26) were performed. Compared with CK-26, CK-21 had 109 up-regulated DEGs and 216 down-regulated DEGs. According to the COG approach, the major DEGs were classified into the COG category S (function unknown) or were unable to be classified into the COG categories. The known functional categories containing the highest amount of up-regulated DEGs were T (signal transduction mechanisms) and O (posttranslational modification, protein turnover, chaperones), and the one containing the highest amount of down-regulated DEGs was K (transcription), which indicated that the low temperature actively induced signal-based transcriptional regulation in *G. uralensis* roots. As shown in Fig. 6b, to investigate why low-temperature roots had significantly higher contents of ILQ and GL when the plantlets were watered by 330 mL/pot/we than those by 270 mL/pot/we, the transcriptome comparisons of [21H-CK v.s. 21L-CK] and [21H-GUH21 v.s. 21L-GUH21] were processed, followed by the DEG overlap identification. It was found that, in comparison with the transcriptomes of 21L-CK and 21L-GUH21, 21H-CK and 21H-GUH21 shared 139 up-regulated DEGs and 227 down-regulated DEGs. Without considering the ones categorized in S and “others,” the up-regulated DEGs categorized in K and T and the down-regulated DEGs classified in K and G (carbohydrate transport and metabolism) were dominant, which suggested that watering actively elicited the plant transcriptional regulation and repressed the carbohydrate metabolism. As shown in Fig. 6c, to identify *G. uralensis* genes significantly influenced by *R. rhizolycopersici* GUH21 to enhance the *G. uralensis* root-associated ILQ accumulation, the transcriptome comparisons of [21H-GUH21 v.s. 21H-CK], [26H-GUH21 v.s. 26H-CK], and [26L-GUH21 v.s. 26L-CK], followed by the DEG overlap identification were performed. It was found that three gene transcripts were significantly up-regulated in ##-GUH21 groups than those in the responding CK groups, including *Glyur000445s00019997*, *Glyur002303s00038706*, and *Glyur000872s00034603*. Among them, *Glyur002303s00038706* encoding a copia-type retrotransposon and *Glyur000872s00034603* encoding proline-rich cell water protein were identified as the down-regulated DEGs in [21L-GUH21 v.s. 21L-CK]. This trend coincided with the ILQ content data in Fig. 5g, suggesting that *Glyur002303s00038706* and *Glyur000872s00034603* might be key genes involved in *R. rhizolycopersici* GUH21-induced ILQ accumulation. On the other hand, five genes were found to be significantly down-regulated in 21H-GUH21, 26H-GUH21, and 26L-GUH21 than those in the responding CK groups, including *Glyur004591s00045196*, *MSTRG.8617*, *Glyur000020s00001789*, *Glyur000966s00025849*, and *Glyur000270s00013309*. Among them, *Glyur000966s00025849* expressing aldehyde dehydrogenase (NAD^+^) and *Glyur000270s00013309* encoding the pathogenesis-related protein 1 (PR1) might be key genes involved in *R. rhizolycopersici* GUH21-induced ILQ accumulation, because they were significantly up-regulated and down-regulated, respectively, in 21L-GUH21 than that in 21L-CK. All above DEG analyses indicated that the significant improvement of secondary metabolism in 21H-GUH21 was due to a complicated metabolic regulation, and thus the regulatory network was predicted based on the detailed analysis in Discussion.

## Discussion

**Fig. 7:**
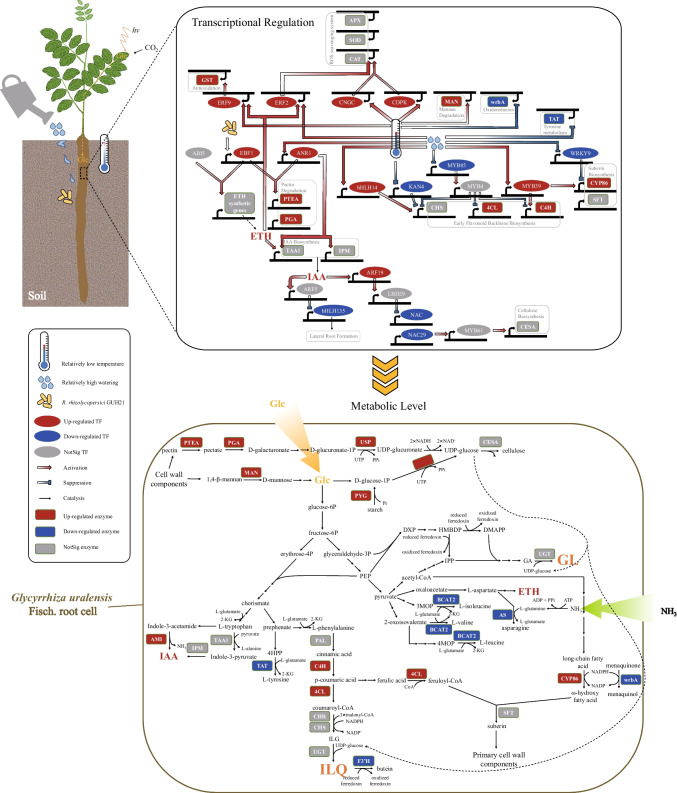
The diagrams showing the integrated effects of the relatively low temperature, the relatively high-level watering, and *R. rhizolycopersici* GUH21 on ILQ and GL-related transcriptional regulation (upper) and metabolism (lower) in the 60-day-old *G. uralensis* roots. IAA, indole-3-acetate; ETH, ethylene; DXP, 1-deoxy-d-xylulose 5-phosphate; HMBDP, 1-hydroxy-2-methyl-2-(E)-butenyl-4-diphosphate; GA, glycyrrhetinic acid; IPP, isopentenyl diphosphate; DMAPP, dimethylallyl diphosphate; 3MOP, (S)-3-Methyl-2-oxopentanoic acid; 4MOP, 4-methyl-2-oxopentanoic acid; 2-KG, 2-ketoglutarate; 4HPP, 4-hydroxyphenylpyruvate; PAL, phenylalanine ammonia-lyase; C4H, cinnamate 4-hydroxylase; 4CL, 4-coumarate CoA ligase; CHR, chalcone reductase; CHS, chalcone synthase; UGT, UDP-glucosyltransferase; BCAT2, branched-chain amino acid aminotransferase 2; AS, asparagine synthetase; PTEA, pectinesterase; PGA, polygalacturonase; MAN, mannan endo-1,4-beta-mannosidase; USP, UDP-sugar pyrophosphorylase; SFT, suberin feruloyl transferase; AMI, amidase; TAA1, L-tryptophan, pyruvate aminotransferase; IPM, indole-2-pyruvate monooxygenase; TAT, tyrosine aminotransferase; PYG, Alpha-1,4 glucan phosphorylase; GUS, β-glucuronidase; CESA, cellulose synthase; F3′H, flavonoid 3′-monooxygenase; wrbA, NADPH dehydrogenase

In our study, the bioinformatic analysis of the 42 *G. uralensis* root-associated flavonoid profiles and their responding edaphic factors and endophytic bacterial communities suggested that soil moisture and soil temperature were key abiotic factors to influence the root-associated ILQ accumulation (Table 2), and the influences might be partially performed through some endophytes (Fig. 2c and Fig. 3). The integrated effect of temperature, soil moisture, and one key endophyte *R. rhizolycopersici* GUH21 on *G. uralensis* root-associated flavonoid accumulation was confirmed in the pot experiment (Fig. 4g). Interestingly, the enhancement of GL production was also observed in the experimental seedlings (Fig. 4h), being in agreement with the study by Yu et al. (2015), which reported that GL content was positively correlated with flavonoid content in the 2-year-old *G. uralensis* roots. The differential gene expression analysis of the experimental and control *G. uralensis* roots indicated that the significant improvement of secondary metabolism was due to a complicated transcriptional regulation. Considering that transcription factors (TFs) played the key roles during the plant transcriptional regulation, the TF-encoding DEGs being associated with secondary metabolism were paid much attention to. Their relationships with the upstream environmental elicitors (i.e., bacterial inoculum, temperature, and watering) and the downstream enzyme-encoding genes were drawn in Fig. 7 according to previous reports and our inference as described in the following paragraphs. Generally, it was inferred that both the relatively high watering and the relatively low temperature should respond for the enhanced secondary metabolite backbone production through their hierarchical transcription activation of the flavonoid biosynthetic genes and the redox regulation genes, while both *R. rhizolycopersici* GUH21 and the relatively high-level watering might respond for the recharge of UDP-glucose, which could be added on the secondary metabolite backbones to synthesize *in planta* bioactive glycosides, through their indirect inhibition of the secondary cell wall polysaccharide production and the lateral root formation in *G. uralensis* root cells. The detailed ratiocination to support the inference is described as follows.

### Improvement of the aglycone biosynthesis in *G. uralensis* roots via the relatively low temperature

In our study, among the relatively low-temperature-induced DEGs between 21-CK and 26-CK (Fig. 6a), there was one TF-encoding DEG being associated with flavonoid biosynthesis *Glyur000281s00018236*, which encoded a GAPR family transcriptional suppressor KANADI4 (KAN4) of early and late flavonoid biosynthetic genes such as chalcone synthase (CHS) (Gao et al. 2010). As shown in Fig. 7, it was down-regulated in 21-CK, suggesting that the transcriptional suppression of KAN4 on CHS was weakened under the relatively low temperature. For the up-regulated DEGs induced by the low temperature, even though there was no secondary metabolism-associated TF-encoding DEGs, several genes involved in the redox regulation were identified, including *MSTRG.335*, which encoded cyclic nucleotide gated channel (CNGC) with the function of the pattern-triggered and effector-triggered immunity mediation in plants (Zhao et al. 2021), *Glyur000254s00017611*, which encoded calcium-dependent protein kinase (CDPK) with the function of increasing plant cold tolerance and root lengths by activating the reactive oxygen species (ROS)-scavenging and stress-related genes (Dong et al. 2020), and *Glyur000014s00002589*, which encoded ethylene-responsive transcription factor 9 (ERF9) with the function to activate the transcription of glutathione S-transferase (GST)-encoding genes to detoxicate the oxidative stress (Zhang et al. 2022). Meanwhile, NAD(P)H dehydrogenase wrbA-encoding gene *Glyur002081s00034191*, which catalyzed the oxidation of menaquinone via NAD(P)H, was significantly down-regulated in CK-21 (Fig. 7). Considering reduced flavodoxins could work as the electron carriers by cytochrome P450s for the production of bioactive natural products (Mellor et al. 2019), the up-regulation of redox-regulating TF-encoding genes and the down-regulation of *wrbA* were reasonably associated with ILQ and GL accumulation in *G. uralensis* roots under the relatively low temperature in our study.

### Inducement of the glucosyl unit accumulation and flavonoid backbone production in *G. uralensis* root cells via the relatively high-level watering

For the high-level watering-associated DEGs (Fig. 6b), there were 13 down-regulated and 4 up-regulated TF-encoding DEGs in both 21H-CK and 21H-GUH21, indicating that the activity of transcription regulation in the seedlings might decrease when they were faced with the relatively high-level watering. Interestingly, among the 13 down-regulated TF-encoding DEGs, nine genes were associated with drought and salinity stress responses via regulating the primary and/or secondary cell wall constituent biosynthesis: *Glyur000821s00020788* encoded WRKY9, the salt-induced transcriptional suppressor of suberin monomer biosynthetic genes to reduce the suberin lamellae in the inner face of the primary cell walls (Krishnamurthy et al. 2021); *Glyur000764s00026022* encoded WRKY47, the transcriptional activator of genes expressing extensin-like protein and wall-bound xyloglucan hydrolase, whose overexpression could increase the plant mineral tolerance (Li et al.); *Glyur006491s00044883* and *Glyur000933s00028081* encoded the drought-, salt-, and/or phytohormone-inducible ERFs, being involved in the secondary cell wall biosynthesis and metabolic regulation (Zhai et al. 2013; Dharanishanthi and Ghosh Dasgupta 2018); *Glyur006571s00047023*, *Glyur000020s00001828*, and *Glyur000844s00023273* encoded NAC-domain containing proteins, which were reported to be the top-layer TFs for secondary wall formation (Zhong and Ye 2014); *Glyur000022s00002063* encoded NAC29-like, the phytohormone-induced transcriptional activator of MYB61 and then the secondary cell wall cellulose synthase gene (Huang et al. 2015); *Glyur001506s00037429* encoded MYB85, the transcriptional activator of the lignin biosynthetic genes during secondary cell wall formation and the TF gene *MYB4* which could specifically inhibit flavonoid biosynthesis (Geng et al. 2020). Meanwhile, as shown in Fig. 7, several genes involved in pectin and starch degradation, such as *Glyur000490s00023911* encoding polygalacturonase (PGA) and *Glyur004265s00037613* encoding α-1,4 glucan phosphorylase (PYG), were found to be significantly up-regulated in both 21H-CK and 21H-GUH21. Ding et al. (2019) found that the promoters of cell wall degradation genes in papaya, such as pectinesterase (PTEA)-encoding genes and PGA-encoding genes, were transcriptional activated by the interaction of CpEBF1 and CpMADS1/3. Coincidentally, in our study, the transcription of MADS-box transcription factor (ANR1)-encoding gene *Glyur000270s00013267* and the ethylene-intensitive3 transcriptional factor (EIN3) binding F-box protein 1 (EBF1)-encoding gene *Glyur000445s00019997* were up-regulated by high watering and *R. rhizolycopersici* GUH21, respectively (Fig. 7). Meanwhile, it was inferred that cellulose biosynthesis was suppressed in 21H-CK and 21H-GUH21 groups, since their top-layer transcriptional repressor IAA (Lee et al. 2019) was highly produced according to the following four facts: (1) the IAA synthetic gene *Glyur000692s00016543* which encoded amidase (AMI) in tryptophan pathway was significantly up-regulated in both 21H-CK and 21H-GUH21; (2) *Glyur000270s00013267* encoding ANR1, which could positively influence the transcription of IAA biosynthetic and transport genes in lateral roots (Sun et al. 2018), was significantly up-regulated in both 21H-CK and 21H- GUH21; (3) *Glyur002876s00038790* encoding tyrosine aminotransferase (TAT) in the tyrosine metabolism, which competed with IAA and flavonoid biosynthesis for chorismite precursor, was down-regulated in both 21H-CK and 21H-GUH21; and (4) *Glyur000605s00024921* encoding branched-chain aminotransferase 2 (BCAT2) in valine, leucine, and isoleucine biosynthesis and *Glyur001058s00023536* encoding asparagine synthetase (AS) in alanine, aspartate, and glutamate metabolism, which competed with the phenylalanine, tyrosine, and tryptophan biosynthesis for amino groups, were down-regulated in both 21H-CK and 21H-GUH21 (Fig. 7). Also, the UDP-sugar pyrophosphorylase (USP) (*Glyur001171s00030969*), which could convert D-glucose-1-phosphate to UDP-glucose precursor of ILQ and GL, was significantly up-regulated in both 21H-CK and 21H-GUH21. All above results reflected that, with the integrated effect of *R. rhizolycopersici* GUH21, *G. uralensis* roots tended to perform the plant secondary metabolite glycosidation rather than the cell wall structural polysaccharide production when the plant was not under the water deficit condition.

On the other hand, as shown in Fig. 7, several genes involved in the flavonoid backbone production, such as *Glyur001446s00035041* encoding cinnamate 4-hydroxylase (C4H) and *Glyur000681s00027307* encoding 4-coumarate-CoA ligase (4CL), were up-regulated in both 21H-CK and 21H-GUH21. The up-regulation of these enzymatic DEGs was probably due to the enhanced expression of two TFs, including bHLH14 (*Glyur000099s00014930*) and MYB39-like (*Glyur000423s00024443*), which were transcriptional activators of flavonoid biosynthetic genes, such as C4H and CHS (Zhang et al. 2015; Li et al. 2021). Meanwhile, ERF2 (*Glyur000067s00006346*), a potential transcription activator of ROS scavenging genes (Yang et al. 2021), was significantly up-regulated in both 21H-CK and 21H-GUH21, suggesting that the high watering amount had the function to regulate the redox balance in *G. uralensis* roots. However, the elicitors or upstream TFs of these up-regulated TFs are not clarified yet.

### Promotion of the glucosyl unit accumulation in *G. uralensis* roots through the IAA signal transduction processes via *R. rhizolycopersici* GUH21

For the central DEGs identified to be associated with *R. rhizolycopersici* GUH21 (Fig. 6c), the ones involved in stress responses, including *Glyur000270s00013309*, which might be transactivated by stress-relating TFs to encode the pathogen defense signaling peptide PR1 (Almeida-Silva and Venancio 2022), and *Glyur000966s00025849*, which encoded an important “toxic aldehyde scavenger” in stress response (Carmona-Molero et al. 2021), were significantly down-regulated in ##-GUH21 groups, suggesting that *R. rhizolycopersici* GUH21 was not a pathogen for *G. uralensis*. Meanwhile, similar like the abiotic factor of high-level watering, *R. rhizolycopersici* GUH21 was inferred to promote the ethylene and IAA production in *G. uralensis* roots. This inference was based on the following three facts: (1) *Glyur000445s00019997* encoding EBF1, which was reported to physically interacted with abscisic acid-insensitive 5 (ABI5) to promote ethylene production in tomato and Fenjiao banana (Song et al. 2022), was significantly up-regulated in ##-GUH21 groups; (2) ethylene signal could promote IAA production via the EIN3-based transcriptional activation of auxin biosynthetic genes (He et al. 2011); and (3) *Glyur000020s00001789* encoding bHLH135, a key regulator of lateral root initiation with whose transcription being suppressed by IAA-inducible auxin response factor 5 (ARF5) (Schlereth et al. 2010; Castelain et al. 2012), was down-regulated in ##-GUH21 groups (Fig. 7). Therefore, it is indicated that *R. rhizolycopersici* GUH21 might cooperate with the high-level watering to facilitate the glucosyl unit accumulation in *G. uralensis* roots through the IAA signal transduction processes.

Besides, the transactivation of different retrotransposons, which were genetic sequences in eukaryotes with the transposition capability through the DNA transcription followed by RNA reverse transcription in a new genome site, in *G. uralensis* roots was found to be modulated by *R. rhizolycopersici* GUH21, wherein the long-terminal repeated (LTR) copia-type retrotransposon (*Glyur002303s00038706*) was up-regulated and the long interspersed nuclear element 1 (LINE1) retrotransposon (*MSTRG.8617*) was down-regulated. This indicates that *R. rhizolycopersici* GUH21 should have the potential to facilitate the environmental adaption of *G. uralensis* by inducing the genetic variation manner modulation in cells. Accordingly, LTR retrotransposons appear to be the major transposable element in plants, while LINE1 is the main retroposition player in mammals (Zhu et al. 2016).

### The bi-directional influences between plants and endophytes

Similar with our finding, Stringlis et al. (2018) found that a plant growth-promoting rhizobacterium (PGPR) *Pseudomonas simiae* WCS417 could promote the production of scopolin, a plant-derived coumarin, and the excretion of scopoletin, the aglycone form of scopolin, in roots of the model plant *Arabidopsis thaliana* by triggering the expression of root-specific TF MYB72 and the MYB72-regulated β-glucosidase BGLU42. In turn, the scopoletin exudation was found to inhibit the growth of soil-borne fungal pathogens and preserved *P. simiae* WCS417 in the rhizosphere of *A. thaliana*. These results indicated that *P. simiae* WCS417 and the secondary metabolism of *A. thaliana* had virtuous interactions between each other. Analogously, the influences between *R. rhizolycopersici* GUH21 and the *G. uralensis* secondary metabolism were expected to be bi-directional. Accordingly, some flavonoids secreted from legume roots, such as 7,4′-dihydroxyflavone (Redmond et al. 1986), 2′-O-methylisoliquiritigenin (Masuo Ichimura 1997), naringenin (Novak 2002), and genistein (Li et al. 2016), could recruit soil-borne rhizobia into the plant nodules for the nitrogen fixation under the nitrogen-deficient conditions (Dénarié and Cullimore 1993; Roy et al. 2020). Therefore, it is expected that *G. uralensis* might use its special root flavonoid exudates to recruit the *R. rhizolycopersici* GUH21 inoculum for the root nodulation and then nitrogen fixation to supply more ammonium (NH_4_^+^) for plant growth and secondary metabolism. This hypothesis would be investigated in our further study. Our finding enriches the beneficial plant–microbe interaction types and would be significant for the planting of high-quality medicinal plants in the future.

## Supplementary Information

Below is the link to the electronic supplementary material.

Supplementary file1 (PDF 1078 KB)

## Data Availability

The raw 16S rDNA amplicon data of the root-associated endophytic bacterial communities collected from different places were deposited into the National Microbiology Data Center (NMDC) with the accession number of NMDC10017952 (https://nmdc.cn/resource/genomics/project/detail/NMDC10017952). The RNA-seq raw data of the cultivated *G. uralensis* Fisch. roots were deposited into NMDC with the accession number of NMDC10017992 (https://nmdc.cn/resource/genomics/project/detail/NMDC10017992).
