# Supplementary material for: Abiotic factors and endophytes co-regulate flavone and terpenoid glycoside metabolism in Glycyrrhiza uralensis
Source: Appl Microbiol Biotechnol. 2023 Mar 3;107(7-8):2671–88. doi: 10.1007/s00253-023-12441-3 (PMC10033487; doi:10.1007/s00253-023-12441-3)
Supplement: Supplementary file 1 — Supplementary file1 (PDF 1078 KB) [file 253_2023_12441_MOESM1_ESM.pdf]

Applied Microbiology and Biotechnology

Supplementary file for:

Abiotic factors and endophytes co-regulate flavone and terpenoid glycoside metabolism in *Glycyrrhiza uralensis*

Zidi Liu[a,#], Yunyang Ma[a,#], Xuelian Lv[b], Nannan Li[a], Xiaohan Li[a], Jianmin Xing[c], Chun Li[d*], Bing Hu[a,e*]

[a] Institute of Biochemical Engineering, College of Chemistry and Chemical Engineering, Beijing Institute of Technology, Beijing, 102401, P. R. China

[b] Ningxia Academy of Agriculture and Forestry Sciences, Yinchuan, 750002, P. R. China

[c] CAS Key Laboratory of Green Process and Engineering & State Key Laboratory of Biochemical Engineering, Institute of Process Engineering, Chinese Academy of Sciences, Beijing, 100190, P. R. China

[d] Key Lab for Industrial Biocatalysis, Ministry of Education, Department of Chemical Engineering, Tsinghua University, Beijing, 100084, P. R. China

[e] Key Laboratory of Medical Molecule Science and Pharmaceutical Engineering, Ministry of Industry and Information Technology of China, Beijing, 102401, P. R. China

[#] Zidi Liu and Yunyang Ma contributed equally to this work.

[*] address a first correspondence to Chun Li (lichun@mail.tsinghua.edu.cn) and a second correspondence to Bing Hu (binghu319@bit.edu.cn), with the telephone number of +86-10-8138139.

Methods S1 Determination of the edaphic parameters

The seven edaphic parameters of the sampling soil, including the soil moisture, pH, saltiness, total carbon content (TC), total nitrogen content (TN), organic carbon content (OC), and TC/TN, were determined in our lab. The methods that were used are listed as follows:

Soil moisture: Soil moisture was determined using the difference method. In details, around 2 g fresh soil were weighed to obtain the exact weight m_0, and then the fresh soil was dried in an electric furnace (Shanghai Yiheng Instruments Co., Ltd., Shanghai, P. R. China) at 90℃ for hours and was weighed again to obtain the exact weight m_1. Finally, the soil moisture was calculated according to the following equation:

$$\text{soil water content (\%)} = \frac{m_0 - m_1}{m_0} \times 100\% \qquad (1)$$

pH and saltiness: A weight of 10 g fresh soil was suspended using 500 mL ddH_2O, and then its pH value was measured using a pH probe (Mettler Toledo, Zurich, Switzerland). Afterwards, the suspension was kept at room temperature for 30 min, and then the soil salinity was inferred from the electrical conductivity (EC_a) of the upper phase according to Jiao et al. (Jiao and Lu 2019). A conductivity meter (Meacon Automation Technology Co., Ltd, Hangzhou, P. R. China) was utilized in the process.

TC and TN: A weight of 0.001 g dried soil sample was placed in the elementary analyzer EA3000 (Eurovector, Pavia, Italia) to measure the contents of carbon and nitrogen elements according to Nicolás et al.(Nicolás et al. 2019).

OC: The content of organic carbonaceous macerals in the sampled soil was measured using the acidification and the elementary analysis according to Liu et al. (Liu et al. 2021). In details, around 5 g dried soil was weighed (w_0) and was acidified using 1 mol/L HCl to discard carbonate and bicarbonate. Until no bubble was observed anymore, the processed soil was dried in the electric furnace, and then weighed (w_1) and processed in the elementary analyzer to measure the fraction of carbon (f_c). Finally, the content of OC in dried soil was calculated according to the following equation:

$$\text{OC (mg/g)} = \frac{f_c \times w_1}{w_0} \times 1000 \qquad (2)$$

TC/TN: It was the ratio of TC to TN in each soil sample.

Methods S2 Analysis of root-associated secondary metabolites through HPLC

The extracted ethanol solutions of *G. uralensis* roots were loaded in HPLC (1260 Infinity; Agilent, Santa Clara, CA, United States) containing the C18 reversed-phase HPLC column (E86713: 250 mm × 4.6 mm, 5 μm; Kromasil, Bohus, Sweden). The mobile phase was comprised of solvent A (acetonitrile) and solvent B (0.05% H_3PO_4), and a series of proportional changes between solvents A and B were required during the detection: 0-4 min, 22% A and 78% B; 27 min, 80% A and 20% B; 35 min, 22% A and 78% B; 55 min, 80% A and 20% A; 60 min, 22% A and 78% B. Some other HPLC conditions were set up as follows: the column oven temperature was 40℃, the sample loading amount was 10 uL, the flow rate was 1.0 mL/min, and the detector wavelengths were set as 240 nm for LG, 245 nm for GL, 285 nm for NHDC and 380 nm for LCA, ILQ and ILG.

References

Jiao S, Lu Y (2019) Soil pH and temperature regulate assembly processes of abundant and rare bacterial communities in agricultural ecosystems. Environ Microbiol 22: 1052-1065. doi: 10.1111/1462-2920.14815

Liu Y, Wang H, Peng Z, Li D, Chen W, Jiao S, Wei G (2021) Regulation of root secondary metabolites by partial root‐associated microbiotas under the shaping of licorice ecotypic differentiation in northwest China. J Integr Plant Biol 63: 2093-2109. doi: 10.1111/jipb.13179

Nicolás C, Martin-Bertelsen T, Floudas D, Bentzer J, Smits M, Johansson T, Troein C, Persson P, Tunlid A (2019) The soil organic matter decomposition mechanisms in ectomycorrhizal fungi are tuned for liberating soil organic nitrogen. ISME J 13: 977-988. doi: 10.1038/s41396-018-0331-6

Table S1. The list of culture media used to screen out certain endophytes

Bacterial genus	Proposed medium	Proposed medium composition
Azoarcus<br>*Stenotrophomonas*<br>*Zoogloea*<br>*Tolumonas*	1	Peptone 5.0 g/L, meat extract 3.0 g/L, licorice root powder 0.01 g/L, agar if necessary 15.0 g/L
Sandaracinus	67	Casitone 3.00 g/L, $CaCl_2$•$2H_2O$ 1.36 g/L, yeast extract 1.00 g/L, licorice root powder 0.01 g/L, agar if necessary 15.00 g/L
Rhizobiales family	130	Sucrose 30.0 g/L, $NaNO_3$ 3.0 g/L, $MgSO_4$•$7H_2O$ 0.5 g/L, KCl 0.5 g/L, $FeSO_4$•$7H_2O$ 0.01 g/L, K_2HPO_4 1.0 g/L, licorice root powder 0.01 g/L, agar if necessary 13.0 g/L
Propionivibrio<br>*Magnetospirillum*	385	Na_2SO_4 3.0 g/L, KH_2PO_4 0.2 g/L, NH_4Cl 0.3 g/L, NaCl 7.0 g/L, $MgCl_2$•$6H_2O$ 1.3 g/L, KCl 0.5 g/L, $CaCl_2$•$2H_2O$ 0.15 g/L, Na-benzoate 0.4 g/L, Na-resazurin solution (0.1% w/v) 0.5 mL/L, licorice root powder 0.01 g/L, agar if necessary 15.0 g/L
Iamia	514	Bacto peptone 5.00 g/L, bacto yeast extract 1.00 g/L, Fe(III) citrate 0.10 g/L, NaCl 19.45 g/L, $MgCl_2$ 5.9 g/L, Na_2SO_4 3.24 g/L, $CaCl_2$ 1.80 g/L, KCl 0.55 g/L, $NaHCO_3$ 0.16 g/L, KBr 0.08 g/L, $SrCl_2$ 34.00 mg/L, H_3BO_3 22.00 mg/L, Na-silicate 4.00 mg/L, NaF 2.40 mg/L, $(NH_4)NO_3$

		1.60 mg/L, Na_2HPO_4 8.00 mg/L, licorice root powder 0.01 g/L, agar if necessary 15.00 g/L
Janthinobacterium<br>*Comamonadaceae*<br>*Ottowia*	535	Trypticase soy broth 30.0 g/L, licorice root powder 0.01 g/L, agar if necessary 15.0 g/L
Aquicella	585	Yeast extract 20.0 g/L, ACES (N-2-acetamido-2-aminoethane-sulfonic acid) 20.0 g/L, activated charcoal 4.0 g/L, licorice root powder 0.01 g/L, agar if necessary 15.0 g/L
Arenimonas<br>*Candidatus*<br>*Pelomonas*<br>*Pleomorphomonas*	830	Yeast extract 0.50 g/L, proteose peptone (Difco no. 3) 0.50 g/L, casamino acids 0.50 g/L, glucose 0.50 g/L, soluble starch 0.50 g/L, Na-pyruvate 0.30 g/L, K_2HPO_4 0.30 g/L, $MgSO_4$•$7H_2O$ 0.05 g/L, licorice root powder 0.01 g/L, agar if necessary 15.0 g/L
Clostridium	54b	Glucose 20.0 g/L, yeast extract 10.0 g/L, $CaCO_3$ 20.0 g/L, licorice root powder 0.01 g/L, agar if necessary 17.0 g/L
Syntrophotalea<br>*Citrifermentans*<br>*Rhodocyclaceae* family<br>*Ketobacter*<br>*Methylophilaceae* family<br>*Rhodospirillales* order<br>*Steroidobacteraceae* family<br>*Geminicoccaceae* family	NB	Peptone 10.0 g/L, beef extract 3.0 g/L, NaCl 5.0 g/L, licorice root powder 0.01 g/L, agar if necessary 15.0 g/L

Arenibacter
Rhodomicrobium
Sphingomonadaceae family
Burkholderiales order
Aeromonas
Thermoleophilia class
Nitrosomonadaceae family
Holosporaceae family

Table S2. Edaphic conditions of the sampling sites

	Temperature (℃)	pH	Saltiness (mg/kg) [a]	Moisture (%)	TC (mg/g) [b]	OC (mg/g) [c]	TN (mg/g) [d]	TC/TN
HJQ-C	14.9±0.866	8.237±0.104	9.064±0.287	18.319±0.745	22.82±2.148	19.868±3.465	0.517±0.3	63.129±48.946
HJQ-W	19.3±0.458	8.583±0.078	3.606±0.212	13.208±2.339	10.07±0.459	7.993±3.76	0.127±0.055	89.019±32.853
HSP-C	16.067±0.551	7.947±0.146	3.424±0.498	14.276±0.563	17.06±2.591	11.569±0.83	0.57±0.157	30.665±4.9
HSP-W	22.533±1.436	8.487±0.234	2.323±0.76	2.342±0.734	6.973±0.433	5.322±0.867	0.12±0.017	59.295±12.496
JQ-C	17.3±0.173	7.063±0.097	33.355±5.347	16.067±1.569	24.9±2.182	22.804±1.354	0.217±0.04	119.431±36.254
JQ-W	25.467±12.761	7.19±0.202	21.469±9.418	5.974±4.492	21.727±5.673	18.466±5.973	0.317±0.311	295.092±404.505
MQ-C	19.3±1.375	7.87±0.026	12.713±1.184	9.744±1.043	19.157±3.955	12.288±1.817	0.177±0.012	109.675±28.48
MQ-C7	19.9±0.52	8.23±0.085	3.549±0.226	5.873±1.297	15.69±0.45	12.929±1.516	0.24±0.035	66.181±8.374
YC-C	18.8±0	7.86±0.07	3.224±0.079	6.852±0	20.36±0	14.714±0	0.47±0	43.319±0
YC-W	19.9±0.346	8.143±0.091	3.36±0.415	6.226±1.378	18.747±2.95	14.275±2.875	0.36±0.139	56.552±17.449
YZ-C	14.767±0.231	7.827±0.225	4.285±1.746	12.518±1.392	27.69±4.992	23.759±4.34	0.837±0.481	41.174±22.073
YZ-W	15.667±1.026	8.213±0.068	3.245±0.31	5.933±0.569	23.823±1.353	17.101±5.09	0.327±0.115	78.777±25.283
ZY-C9	16.5±0.173	8.37±0.459	9.333±12.944	8.613±6.551	7.097±3.389	5.365±2.544	0.093±0.058	80.049±9.731
ZY-W	20.767±0.643	8.12±0.303	3.25±0.311	2.861±0.841	5.737±1.279	3.856±0.951	0.04±0.04	76.292±71.025

[a] Saltiness, salt content in dry soil; [b] TC, total carbon content in dry soil; [c] OC, organic carbon content in dry soil; [d] TN, total nitrogen content in dry soil

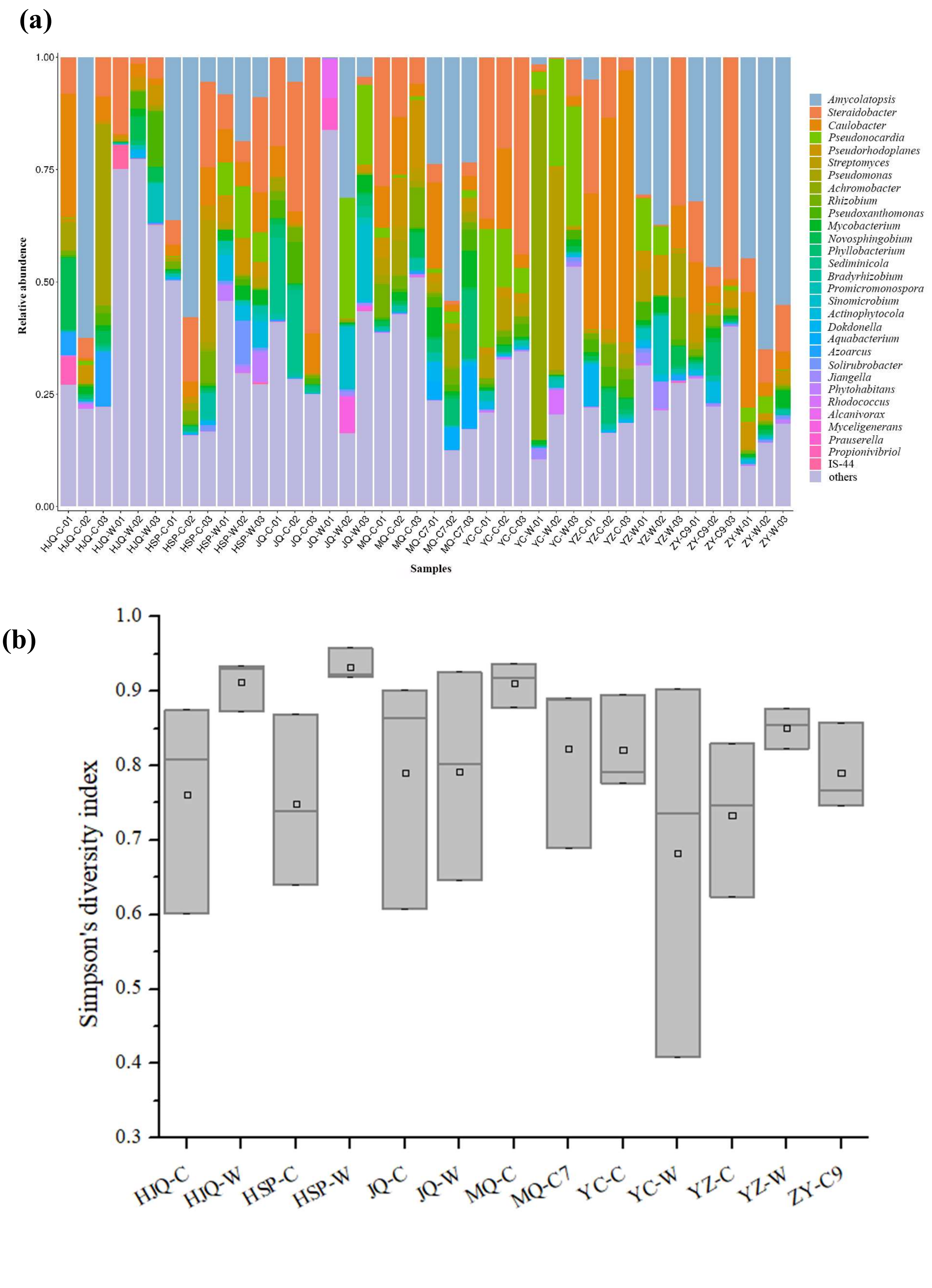
(a)
Relative abundence
Samples
Amycolatopsis
Steraidobacter
Caulobacter
Pseudonocardia
Pseudorhodoplanes
Streptomyces
Pseudomonas
Achromobacter
Rhizobium
Pseudoxanthomonas
Mycobacterium
Novosphingobium
Phyllobacterium
Sediminicola
Bradyrhizobium
Promicromonospora
Sinomicrobium
Actinophytocola
Dokdonella
Aquabacterium
Azoarcus
Solirubrobacter
Jiangella
Phytohabitans
Rhodococcus
Alcanivorax
Myceligenerans
Prauserella
Propionivibriol
IS-44
others
(b)
Simpson's diversity index
HJQ-C
HJQ-W
HSP-C
HSP-W
JQ-C
JQ-W
MQ-C
MQ-C7
YC-C
YC-W
YZ-C
YZ-W
ZY-C9

(c)

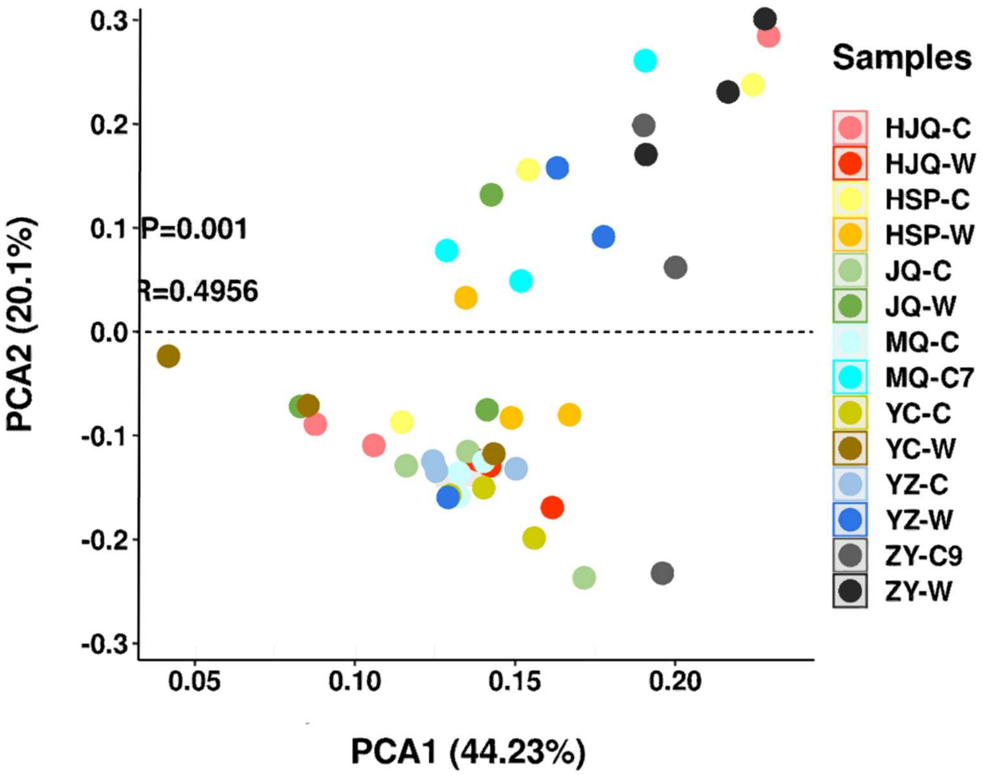

Figure S1. Analysis of the root-associated endophytic bacterial community structures collected from wild and cultivated *Glycyrrhiza uralensis* in distinct places in northwest China. (a) The histogram showing bacterial community compositions in the genus level; (b) Simpson's diversity indices of the endophytic bacterial communities; (c) the principal component analysis (PCA) plot of the bacterial community compositions.
